# Information Structure and Word Order Canonicity in the Comprehension of Spanish Texts: An Eye-Tracking Study

**DOI:** 10.3389/fpsyg.2021.629724

**Published:** 2021-04-06

**Authors:** Carolina A. Gattei, Luis A. París, Diego E. Shalom

**Affiliations:** ^1^Instituto de Física de Buenos Aires (CONICET), Universidad de Buenos Aires, Buenos Aires, Argentina; ^2^Laboratorio de Neurociencia, Universidad Torcuato Di Tella, Buenos Aires, Argentina; ^3^Facultad de Filosofía y Letras, Pontificia Universidad Católica Argentina, Buenos Aires, Argentina; ^4^Grupo de Lingüística y Neurobiología del Lenguaje, Instituto de Ciencias Humanas Sociales y Ambientales (CONICET), Mendoza, Argentina

**Keywords:** information structure, word order, eye-tracking, text comprehension, prominence, psych verbs

## Abstract

Word order alternation has been described as one of the most productive information structure markers and discourse organizers across languages. Psycholinguistic evidence has shown that word order is a crucial cue for argument interpretation. Previous studies about Spanish sentence comprehension have shown greater difficulty to parse sentences that present a word order that does not respect the order of participants of the verb's lexico-semantic structure, irrespective to whether the sentences follow the canonical word order of the language or not. This difficulty has been accounted as the cognitive cost related to the miscomputation of prominence status of the argument that precedes the verb. Nonetheless, the authors only analyzed the use of alternative word orders in isolated sentences, leaving aside the pragmatic motivation of word order alternation. By means of an eye-tracking task, the current study provides further evidence about the role of information structure for the comprehension of sentences with alternative word order and verb type, and sheds light on the interaction between syntax, semantics and pragmatics. We analyzed both “early” and “late” eye-movement measures as well as accuracy and response times to comprehension questions. Results showed an overall influence of information structure reflected in a modulation of late eye-movement measures as well as offline measures like total reading time and questions response time. However, effects related to the miscomputation of prominence status did not fade away when sentences were preceded by a context that led to non-canonical word order of constituents, showing that prominence computation is a core mechanism for argument interpretation, even in sentences preceded by context.

## 1. Introduction

Word order alternation is a frequent feature in many languages across the world. Several works have tried to explain the psycholinguistic principles that govern comprehension of alternative word orders. Based on theoretical accounts of word order alternation or “scrambling,” many of these studies assume the existence of a particular canonical word order for each language (e.g., SVO for English, SOV for German, etc.), and alternative orders derived from it (Comrie, 1989). Experimental evidence suggests that alternative word orders are more difficult to process than canonical ones, as reflected by longer reading times, response times and lower accuracy rates (Hyönä and Hujanen, 1997; Bader and Meng, 1999; Kamide and Mitchell, 1999). Studies about the role of word order for incremental comprehension have also shown that word order alternation is a relevant cue for lexico-semantic argument interpretation and posterior realization of syntax-to-semantics linking. In other words, incremental processing of word order features are useful to predict “*who does what to whom”* in a given event (see Bader and Bayer, 2006; Bornkessel and Schlesewsky, 2006, for two different reviews on this issue). For instance, a Spanish cloze task has shown that while the appearance of a nominative-marked argument in first position leads readers to expect an activity verb, the appearance of a dative-marked argument in first position leads them to expect an Object Experiencer psychological verb (heareafter ObjExp psych verb, Gattei et al., 2015b, Experiment 2). The violation of these expectations generates higher error rates and response times, longer reading times and amount of regressions to previous regions (Gattei et al., 2015a, 2017), and differential neural correlates (Gattei et al., 2015b), even in the canonical word order of the language. The interpretive function of word order has also been evaluated in a spectrum of languages with different degrees of complexity regarding morphological case marking, such as German (Bornkessel et al., 2003, 2005), Italian (Dröge et al., 2014), and Chinese (Wang et al., 2012) with very similar and robust results. Bornkessel and Schlesewsky (2006) suggest that word order—as well as case marking, animacy, and definiteness—are key linguistic features for the computation of argument prominence, which comprises the hierarchical relation among arguments in a sentence (Lamers and De Swart, 2012). The evidence suggests that the human sentence parser tends to interpret the first argument of a sentence as the most “Actor-like” possible according to the prominence status provided by those features. This proposal suggests that speakers tend to compute arguments prominence status by following a more-to-less prominent order, this is, following the stipulated order of arguments in the lexico-semantic structure of verbs. Hence, a Spanish animate, nominative-marked, definite argument in first position will most likely be the Actor of an activity event, and an animate, dative-marked, definite argument in first position will most likely be the Experiencer of a psych state.

However, an aspect that has not been taken into account by most studies that address word order alternation in sentence comprehension is that the appearance of non-canonical word order is not arbitrary but rather motivated by discursive factors like, for instance, if a referent has been previously introduced or if it is part of a referent mentioned before (Givón, 1984; Lambrecht, 1994; Birner and Ward, 1998). Along with prosody, word order is considered one of the key information structure markers across languages. The way in which given and new information is conveyed can modulate pragmatic interpretation by stipulating the status of constituents as discourse topic and focus. For instance, when unmarked, Spanish favors given information in the left-most position of the sentence, even when that means to change from canonical SVO word order to a non-canonical one (Zubizarreta, 1998) as it may be seen in (1):


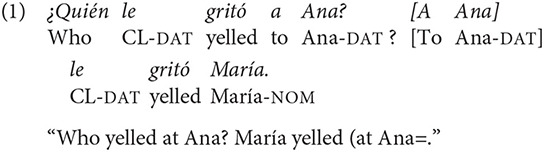


Typologically speaking, Spanish is considered to be a flexible language regarding both the possibility of alternating word order and the lack of constraints about the syntactic positions in which focus can potentially be assigned (Van Valin, 1999; Belloro, 2012). Hence, the same question posited in (1) may present a response in which new (focused) information takes place in first position, as shown in (2).


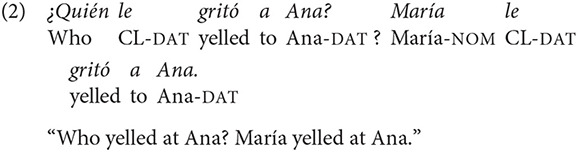


In this example, the response to the question may be interpreted as narrow focus, in the sense that it was María, and not Juan, for instance, who yelled at Ana. Thus, the appearance of new information in first position may modify the way speakers interpret the response. From a psycholinguistic point of view, not many studies have addressed the role of word order for discourse on-going interpretation. In a study about processing of declarative sentences in Finnish with non-canonical information structure Kaiser and Trueswell (2004) argue that readers may need additional presuppositions in order to understand isolated sentences with a non-canonical word order. Hence, showing the right discourse setting for this type of sentences should facilitate comprehension. The authors showed that the presentation of a referent providing new information in first position entailed longer reading times irrespective of word order (SVO vs. OVS), and that overall, sentences with non-canonical word order (OVS) were more difficult to understand that sentences that followed the canonical word order of the language. This means that the presentation of a supportive discourse context partially alleviates the usual difficulty associated to a non-canonical construction.

In a series of Event-Related Potentials (ERPs) experiments in German that manipulated position of the referents and givenness, Schumacher and Hung (2012) showed that new inferred information in sentence-medial positions engender a Late Positivity when compared to new given information. This difference does not take place when the constituents are in sentence-initial position. The authors claim that “information presented in sentence-initial position is treated differently than information in other positions during both language processing,” and the construction of discourse representation structure.

Burmester et al. (2014) also showed that topic-first order eases OVS sentence processing in German-speaking adults, as evidenced by an offline comprehensibility judgment task and a late positivity effect at the ERP mean voltage when comparing sentences preceded by a neutral context and those preceded by a topicalized context.

The present study seeks to go a step forward and to evaluate how the pragmatic use of word order alternation interacts with its use as a cue for arguments prominence computation. In other words, if prominence is considered a hierarchy composed by other independent hierarchies (e.g., animacy features are independent from case marking and word order), and in a particular sentence these hierarchies may conflict with each other (e.g., the innanimate argument bares nominative case, Chow and Phillips, 2013), it is worth exploring when the language word order (SVO) is incongruent with the canonical word order stipulated by the lexico-semantic structure of the verb (SVO for activity verbs and OVS for ObjExp psych verbs) and that of the rhematic hierarchy (“given” referents precede “new” ones).

The paper is organized as follows: We first present a brief description of Spanish word order alternation, with special emphasis on Object Experiencer Psych Verbs (hereafter ObjExp psych verbs) and stipulate the hypotheses and predictions related to the processing of these sentences when embedded in context. We then present an eye-tracking study addressing these issues. Finally, we discuss the results of the current experiment under the light of previous findings.

### 1.1. Word Order Alternation in Spanish

Spanish is rather flexible in terms of word order, although it is argued to be a Subject-Verb-Object (SVO) language (Contreras, 1976; Suñer, 1982; Ocampo, 1995). Take for instance sentences in (3) and (4)


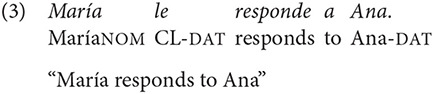



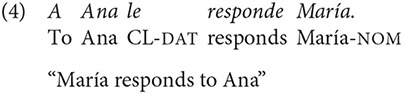


The verb in these examples expresses the same type of event, in which an Actor (“María”) carries out an activity (“to respond”) that affects another participant (“Ana”). The main difference between both sentences is that, apart from showing the canonical word order of the language, sentence (3) shows a canonical order of its arguments, with an Actor preceding the affected participant or “Undergoer” (Foley and Van Valin, 1984). Sentence (4), on the contrary, exhibit both a non-canonical word order and a non-canonical arguments order, with the Undergoer preceding the Actor.

The same morphological case marking is applied to arguments in sentences with ObjExp psych verbs, as shown in (5) and (6)


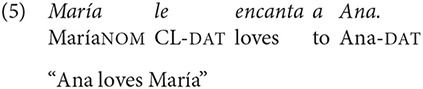



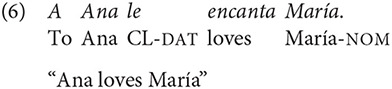


Altough sentence (6) carries a non-canonical word order, it reflects the canonical order of arguments established by its lexico-semantic structure, as exemplified in (7), in which the verb's left-most argument (“x”) is associated to an Experiencer of a state predicate, and “y” is associated to the Theme that generates this state (Van Valin, 2005, p. 45).

(7) encantar'(x,y)

This subclass of psych verbs has become relevant for understanding how the sentence processor uses both syntactic and lexico-semantic information in order to predict the thematic structure of a particular event. In a series of studies run in this language, when presented with sentences like (4) and (6), readers found higher difficulty to integrate the verb and the second argument of the sentence in trials with activity verbs than in sentences with ObjExp psych verbs. The opposite pattern was found for subject-initial sentences, showing longer reading times (Gattei et al., 2015a) and higher amount of regressions (Gattei et al., 2017) to previous regions when the sentence included an ObjExp psych verb than an activity verb.

This pattern of results suggests that readers are not only guided by word order canonicity in order to interpret sentences, but that they use word order together with a semantic principle that stipulates that the first argument will take the most prominent status possible to form predictions about the type of thematic structure that the event will carry and assign a thematic role to the preverbal argument accordingly (Bornkessel et al., 2005; Wolff et al., 2007; Haupt et al., 2008). The appearance of a verb that required a correction of this assumption resulted in longer reading times in the regions that comprised the second argument of the sentence. Furthermore, when asked “who did/felt what for whom” after reading each sentence, accuracy rates were lower and response time longer when the sentences arguments did not reflect the canonical order of arguments of their lexico-semantic structure, showing that the effects of not respecting the order established by the lexico-semantic structure of the verb are so robust that can persist even once all the processes of linguistic integration have been completed.

### 1.2. Hypotheses and Predictions

By means of an eye-tracking reading task we aim at weighing the relative processing load imposed by the violation of two types of linguistic hierarchies related to word order alternation: the rhematic hierarchy—given referents precede new referents (Contreras, 1976)—and that related to arguments' prominence—“the Actor precedes the Undergoer” (Van Valin and LaPolla, 1997).

We propose to replicate the findings from the study of Kaiser and Trueswell (2004), but using two different verb types (i.e., activity verbs and ObjExp psych verbs), as in Gattei et al. (2015a, 2017).

Following the results of Kaiser and Trueswell (2004) and basing our hypotheses on the assumption that word order alternation is motivated by discursive factors (Givón, 1984; Lambrecht, 1994; Birner and Ward, 1998) we expect that overall, the appearance of an adequate context facilitates sentence comprehension. In the current study, context adequacy is provided by the pragmatic status of referents. This means that an adequate context will lead to a sentence with a “given” referent in first position and a “new” referent as second argument, giving rise to a canonical rhematic hierarchy. Conversely, an inadequate context will give rise to a “new” referent in first position and a non-canonical information structure.

We also expect that all effects related to the interaction between syntactic, semantic and pragmatic factors are reflected in late eye-movement measures, since they are assumed to reflect later parsing stages (see Clifton et al., 2007; Vasishth et al., 2013, for a review on this discussion).

In relation to the interaction between both prominence and rhematic hierarchies, we predict two possible outcomes:

1. Context adequacy causes possible effects of prominence miscomputation fade away. The rationale of this prediction is that thematic reanalysis effects found in previous studies could have been the result of making additional presuppositions related to the use of a non-canonical word order without any previous context. This hypothesis predicts a main effect of information structure, and a triple interaction between word order, verb type and information structure, with sentences with non-canonical word order showing higher processing demand when an unsupportive context is used than when preceded with a supportive context. When a supportive context is used, prominence miscomputation effects should disappear. This interaction should take place once the verbs are read and in subsequent regions.
2. Context adequacy plays a role at initial stages of sentence processing but does not make the effect of prominence miscomputation fade away. The rationale of this prediction is that the relation between syntax-to-semantics linking and word order involves a mechanism -semantic roles and syntactic functions- that belongs to the grammatical nucleus of any given language. Thus, the violation of the prominence hierarchy comprises the alteration of a core relationship in a sentence. On the contrary, the relationship between a non-canonical rhematic structure and non-canonical word order involves the manipulation of a more flexible system (i.e., Pragmatics), designed to adapt linguistic form to the dynamics of context. In other words, this hypothesis predicts greater difficulty for sentences with unsupportive context than for those with supportive one. This difficulty should be reflected at the initial regions of sentences (i.e., where the new referent takes place). The hypothesis also predicts higher processing demands for those sentences that do not respect the prominence hierarchy than for those that respect it (i.e., for SVO sentences with ObjExp psych verbs and for OVS sentences with Activity verbs) irrespective of whether they are preceded by a supportive or unsupportive context. Following Gattei et al. (2017), effects of prominence miscomputation should take place at late eye-movement measures at later regions of the sentence (i.e., verb region onward for reading measures, and at initial regions of the sentence and verb for regression measures).

## 2. Materials and Methods

We designed a text reading task using the eye-tracking method in order to study the interaction between word order, verb type and information structure. This technique allows to register with great temporal precision what eyes do during naturalistic reading, and what strategies readers use in order to overcome cognitive difficulties that could arise from linguistic complexity (also see Just and Carpenter, 1980; Just et al., 1982, for a discussion on the advantages of this paradigm).

### 2.1. Participants

Seventy-two native Spanish speakers (47 female, age range 18–54 years old; *M* = 22.6, *SE* = 0.74) participated in this study. All participants had normal or corrected-to-normal vision and had no history of prior neurological disease, drug or alcohol abuse, psychiatric disorders, developmental speech/language disorders, or learning disabilities. All of them provided written consent prior to the study. Sixty-nine of the participants entered the final data analysis, the remaining three having been excluded on the basis of equipment-related artifacts and/or insufficient accuracy in the comprehension task (an error rate higher than 40% in the critical conditions). All participants were compensated with 150 Argentinian Pesos (approximately US$ 9 at that time) after finishing the experiment session.

### 2.2. Materials

A total of 384 texts were built following the studies of Gattei et al. (2017) and Kaiser and Trueswell (2004). The texts consisted of three sentences [hereafter S_1_ refers to the first sentence, S_2_ refers to the second sentence and S_3_, to the third sentence of the text, see example (8)]. S_1_ introduced the first referent (R_1_: Richard) and the situation in which s/he was. S_2_ introduced the second referent (R_2_: Mary/Ana) and stated that this person was performing an action with a person whose name has not been mentioned (R_3_: Ana/Mary). S_3_ comprised the target sentence, which described that R_1_ (Richard) saw or heard that one of the two referents introduced in S_2_ did or felt something for the other person. Sentences were built in such way that R_1_ always had a different gender than R_2_ and R_3_. This was done in order to avoid possible ambiguity in the use of pronouns (“he” or “she”) in the text, so that it was always clear for the reader that it referred to R_1_. In other words, if R_1_ was feminine, then R_2_ and R_3_ were masculine proper names and vice versa.


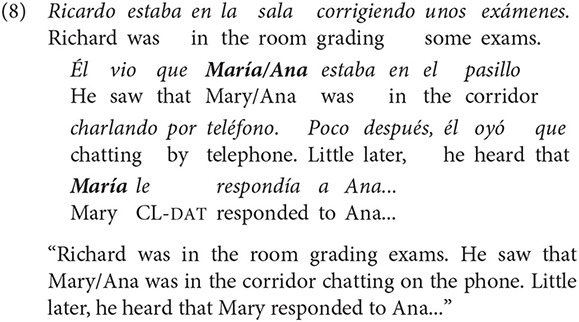


In S_3_, 24 ObjExp psych verbs and 24 activity verbs with dative-marked objects were used. Both verb groups were matched according to length (ObjExp: *M* = 6.8, *SE* = 0.31; Act: *M* = 6.3, *SE* = 0.25) and log-transformed frequency (ObjExp: *M* = 4.32, *SE* = 0.17; Act: *M* = 4.47, *SE* = 0.11) according to the LEXESP database (Davis and Perea, 2005). An independent samples *t*-test revealed that there were no significant differences between groups [Length: *t*_(46)_ = −1.35, *p* > 0.05; log Frequency: *t*_(46)_ = 0.71, *p* > 0.05].

Verbs from S_3_ were framed between a Noun Phrase (NP) and a Prepositional Phrase (PP) that consisted of 48 pairs of proper names matched in length and counterbalanced in gender (half masculine and half feminine). Target sentences could also follow the Subject-Verb-Object (SVO) order or the Object-Verb-Subject (OVS) word order. In this way, we tested the role of constituents order for these types of sentences. Finally, information structure of S_3_ was also manipulated. In four of the target sentences, the referent that appeared in first position in S_3_ had already been mentioned in S_2_, while in the other four, the referent that appeared in first position in S_3_ had not been previously mentioned by its proper name. This means that the configuration of half of the sentences' information structure comprised a given referent in first position while in the other half, a new referent was provided at sentence-initial position.

The 384 total sentences were divided into eight lists of 48 sentences each (six per condition) so that participants would see each verb twice, each time in a sentence with different word order and framed by two different pairs of proper names and a different context.

In order to avoid *wrap-up* effects (Just and Carpenter, 1980), additional phrases were added at the end of S_3_ so that regions of interest did not coincide with the last word of the text. These phrases were semantically neutral so that they would not facilitate the interpretation of S_3_'s argument structure. In order to facilitate the posterior statistical analysis, both the syntactic structure and length of the first two sentences of the text were kept constant among the 48 sets, with a length range between 35 and 52 characters in S_1_ (*M* = 42.5 characters), and 29–40 characters in S_2_ (*M* = 35.42 characters). Length of S_3_ would only vary according to the length of the additional phrase used in order to avoid “wrap-up” effects, with a length range of 75–88 characters (*M* = 82.33 characters).

In addition, a set of three practice items and 72 filler texts that were unrelated to the purposes of the study were used. The latter texts included sentences with different syntactic complexity and length to the target texts, and referred to diverse semantic topics, so that participants could not realize what the main purpose of the study was.

Finally, 123 questions were designed in order to test the comprehension of each practice, critical and filler item. Questions for the critical items were formulated in two ways: In order to respond 32 of the questions, participants had to retrieve the argument structure of the target sentence (S_3_) and participants had to respond whether one of the referents did / felt what for the other referent, while in the remaining 16 texts the question tested the comprehension of one of the two previous sentences (S_1_ and S_2_). The rationale of doing this was to assure that participants would read the context previous to the target sentence. Half of the questions were responded affirmatively and half of them were responded negatively. Half of the questions that referred to S_3_ asked about the subject constituent and half of them referred to the object constituent. Participants had to choose the correct answer by clicking on it with the mouse. Position of the correct answer was half of the times on the right side of the screen and was randomly assigned between trials for each participant. Table 1 shows an example of one of the 48 sets of 8 texts used in the current experiment. A complete list of the experiment materials may be found at Appendix A of the Supplemental Material, available at https://osf.io/kp4dn/.

**Table 1:** Critical sentences used in the current eye-tracking study.

**Condition**	**Context**	**Critical sentence**	**Question**
(a) ObjExp SVO G-N	Ricardo estaba en la sala corrigiendo unos exámenes. Él vio que María estaba en el pasillo charlando por teléfono. Poco después él oyó que.	María le encantaba a Ana aunque no estuvieran de acuerdo.	¿Es María quien le encantaba a alguien?
		*Ana loves Mary although they wouldn't agree*.	
(b) ObjExp OVS G-N		A María le encantaba Ana aunque no estuvieran de acuerdo.	Is it Mary who is loved by someone?
		*Ana loved Mary although they wouldn't agree*.	
(c) Act SVO G-N	*Richard was at the room grading exams. He saw María was at the corridor talking on the phone. Later he heard that*.	María le respondía a Ana aunque no estuvieran de acuerdo.	¿Es María quien le respondía a alguien?
		*Mary responded to Ana although they wouldn't agree*.	
(d) Act OVS N-G		A María le respondía Ana aunque no estuvieran de acuerdo.	Is it Mary who responded to someone?
		*Ana responded to Mary although they wouldn't agree*	
(d) ObjExp SVO N-G	Ricardo estaba en la sala corrigiendo unos exámenes. Él vio que Ana estaba en el pasillo charlando por teléfono. Poco después él oyó que.	María le encantaba a Ana aunque no estuvieran de acuerdo.	¿Es María quien le encantaba a alguien?
		Ana loves Mary although they wouldn't agree.	
(e) ObjExp OVS N-G		A María le encantaba Ana aunque no estuvieran de acuerdo.	Is it Mary who is loved by someone?
		*Ana loved Mary although they wouldn't agree*.	
(f) Act SVO N-G	*Richard was at the room grading exams. He saw Ana was at the corridor talking on the phone. Later he heard that*.	María le respondía a Ana aunque no estuvieran de acuerdo.	¿Es María quien le respondía a alguien?
		*Mary responded to Ana although they wouldn't agree*.	
(g) Act OVS N-G		A María le respondía Ana aunque no estuvieran de acuerdo.	Is it Mary who responded to someone?
		*Ana responded to Mary although they wouldn't agree*	

*ObjExp, Object Experiencer Psych Verbs; Act, Activity Verbs; SVO, Subject-Verb-Object, OVS, Object-Verb-Subject; G-N, Given-New; N-G, New-Given*.

### 2.3. Equipment

Similarly to (Gattei et al., 2017), participants were seated in front of a 19-inch screen (Samsung SyncMaster 997 MB, 1024 × 768 pixels resolution, 100 Hz refresh rate) at a viewing distance of 65 cm. Head movements were prevented with a chinrest aligned with the center of the screen. Gaze locations of both eyes during reading was recorded with an EyeLink 1000 eye-tracker (SR Research Ltd.) at a sampling rate of 1 kHz. As given by the manufacturer, nominal average accuracy was 0.5 ° and space resolution was 0.01° root mean square (RMS). A standard 13-point grid for both eyes was used to calibrate participant's gaze. All recordings and calibration were binocular but only left eye data were used for the analysis.

All eye movements were labeled as fixations, saccades and blinks by the eye-tracker software using the default thresholds for Cognitive experiments (30°/s for velocity, 8,000°/s for acceleration, and 0.1° for motion, Cornelissen et al., 2002). Stimuli presentation was developed using Matlab (http://www.mathworks.com/, Massachusetts, United States) and Psychophysics Toolbox Version 3.

### 2.4. Procedure

All texts were displayed on five lines, the critical sentence being displayed on the fourth line. Neither the first nor the last word of each line displayed any of the main regions of interest from the critical sentence nor any of the referents from S_2_.

In sum, the design of the text was such that: (i) the critical sentence did not exceed one line of the text; (ii) the line of the critical sentence was always the same across trials (line four); and (iii) the line of the critical sentence never started or ended with a critical word.

Sentences were presented in Courier New Bold font. At a distance of 65 cm, each letter subtended 0.44° of visual angle laterally. Subjects were instructed to read the texts at their own rate. No instructions were given to suppress eye blinks.

Before the eye-tracking experiment began, they had a practice session of three texts. At the beginning of each trial, a dot appeared at the top left edge of the screen and after participants fixated on this dot, the text appeared. The first letter of the text was located at the position of the dot. Participants were instructed to look at a second dot at the bottom right corner of the screen to indicate they had finished reading. The total reading time of each trial was measured starting from when participants triggered the appearance of the text by fixating on the left dot until they fixated on the bottom right dot and the text disappeared. Comprehension questions appeared after every text. Participants responded by mouse-clicking on one of two possible answers (“Yes” or “No”) displayed horizontally. Response time was measured starting from the appearance of the question until participants clicked on one of the possible responses. A calibration procedure was performed at the beginning of the eye-tracking experiment. Experimental sessions lasted approximately 45 min.

### 2.5. Data Analysis

Eye movement data from the 69 participants was screened for blinks and track losses. Fixations shorter than 50 ms and longer than 1,000 ms were removed from the analysis. After this screening process, fixations were assigned to their respective word and line. Boundaries between words (x axis) were set by splitting the space between two words in half. Boundaries between lines (y axis) were set by splitting the space between two lines in half. Upper and lower boundaries of the first and last lines were calculated so that they were symmetrical with the lower and upper boundaries of these lines, respectively. Fixations that fell outside the boundaries of the text were eliminated whenever participants continued reading after fixating outside the text area.

Visual inspection was carried out for each trial by providing a number to each fixation and a line that linked consecutive fixations. With this representation it could be easily established whether participants were reading the whole text. Trials in which participants skipped sentences from the context or the critical sentence were erased. Whenever there was a vertical misalignment between the fixations and the lines they belonged to, manual correction of fixations was performed by taking into account the trajectory of the reading path and realigning the fixation to the correct line. Visual inspection and subsequent correction resulted in the removal of 5,293 fixations (0.79% of the data) and realignment of 17537 fixations (2.62%). Besides, 44 trials were removed due to track loss, the appearance of a random reading pattern, or incomplete text reading. This comprises 0.5% of the total data.

Eye-tracking measures were computed using em2 package for R language for statistical computing (Logacev and Vasishth, 2013, version 3.0.2).

For the purpose of analysis, we divided the sentences into ten regions that consisted of the first ten words of each sentence, as shown in Table 2. Note that in order to facilitate statistical analysis and visual presentation of the results, we aligned the critical regions that comprised the proper names (regions 2 and 6), the clitic (region 3) and the verb (region 4). The region of the preposition has been labeled as (5) in subject-initial sentences, and (1) in object-initial sentences. The regions “PP1,” “PP2,” “PP3,” and “PP4” correspond to the first to the fourth word of the prepositional phrase following the second noun phrase.

**Table 2:** Regions of interest used for the statistical analysis of the current eye-tracking experiment according to Word Order.

	**1**	**2**	**3**	**4**	**5**	**6**	**7**	**8**	**9**	**10**
0]*SVO		María	le	respondía|encantaba	a	Ana	PP1	PP2	PP3	PP4
		*Mary*	*clitic*_[*DAT*]_	*responded*|*loved*	*to*	*Ana*_[*DAT*]_	*PP1*	*PP2*	*PP3*	*PP4*
0]*OVS	A	María	le	respondía|encantaba		Ana	PP1	PP2	*PP3*	*PP4*
	*To*	*Mary*_[*DAT*]_	*clitic*_[*DAT*]_	*responded*|*loved*		*Ana*	*PP1*	*PP2*	*PP3*	*PP4*

*SVO, Subject-Verb-Object; OVS, Object-Verb-Subject; PP1, First Word of the Prepositional Phrase; PP2, Second Word of the Prepositional Phrase; PP3, Third Word of the Prepositional Phrase; PP4, Fourth Word of the Prepositional Phrase*.

For each fixated word, we computed the following measures: (1) *First Fixation Duration* (FFD; the duration of the first fixation on the word); (2) *First Pass Reading Time* (FPRT; the sum of all fixation durations on the word before any other word was fixated); (3) *Regression Path Duration* (RPD; also known as *go-past time*, it is the sum of all first-pass fixation durations on the word and all preceding words in the time period between the first fixation on the word up to the point where the reader leaves the critical region with a progressive saccade; (4) *Right-Bounded Regression Count* (RBRC; the number of regressions from the word before any word further to the right has been fixated); (5) *Total Fixation Time* (TFT; the sum of all fixations durations on a word); and (6) *Total Incoming Regressions* (TIR; the number of regressions to a specific word). Measures 1–2 are typically considered early measures, whereas measures 3–6 are considered late measures (Clifton et al., 2007; Vasishth et al., 2013).

Data analysis was conducted in the R programming environment (R Core Team, 2013). For measures comprising reading or response time (i.e., Comprehension Task Response Time, FFD, FPRT, RPD, and TFT) a linear mixed-effects model was fit to the data using the package lme4 (Pinheiro and Bates, 2000; Bates et al., 2014). For the accuracy measure, the data was fit to a generalized linear mixed-effects model with a binomial function, which is adequate for analyzing data measured on a dichotomous scale, namely “Correct” and “Incorrect” response. Count data (RBRC and TIR), on the other hand, was analyzed with a generalized mixed-effects model with Poisson link function, which is appropriate for counts of events in a fixed time window (Baayen, 2008, p. 322).

For the regression models, Verb Type, Word Order and Information Structure were considered fixed effects and Subject, and Item were fit as random effects. Log Frequency and inverse length of each word were included as control factors in every region except for regions 1 and 5 (preposition “a”) and region 3 (clitic). These two variables may explain a significant part of the variability in reading times and amount of fixations on these regions (Just and Carpenter, 1980; Rayner and Well, 1996; Kliegl et al., 2004). As for collinearity between both factors, model comparison among models that included one, the other or both were significantly different. AIC values indicated that models where both factors were included were significantly better than the other two. In consequence, the two of them were included.

A maximal random-effects structure was included in both LMMs and GLMMs whenever it was possible, as linear mixed-effects models that do not consider random intercepts and slopes involve the risk of Type I error inflation (Barr et al., 2013). When models either did not converge or the correlation between variance components could not be estimated, the random effects structure was simplified by removing the correlations. For large samples like the ones collected in this study, the *t* distribution approximates the normal distribution and an absolute value of *t* larger than 2 indicates a significant effect at α = 0.05. For all the models presented in the study, covariates that involved reading time were scaled and centered.

Finally, we used an orthogonal contrast coding to test the interactions among verb type, word order and information structure at the pertinent regions. For the verb type contrast, sentences with activity verbs were coded as −1 and sentences with ObjExp psych verbs were coded as 1. For the word order contrast, SVO sentences were coded as −1 and OVS sentences were coded as 1. Finally, for the information structure contrast, sentences with a new referent in first position were coded as −1 and sentences with a given referent in first position were coded as 1.

## 3. Results

### 3.1. Comprehension Task

#### 3.1.1. Total Reading Time

**Figure 1:**
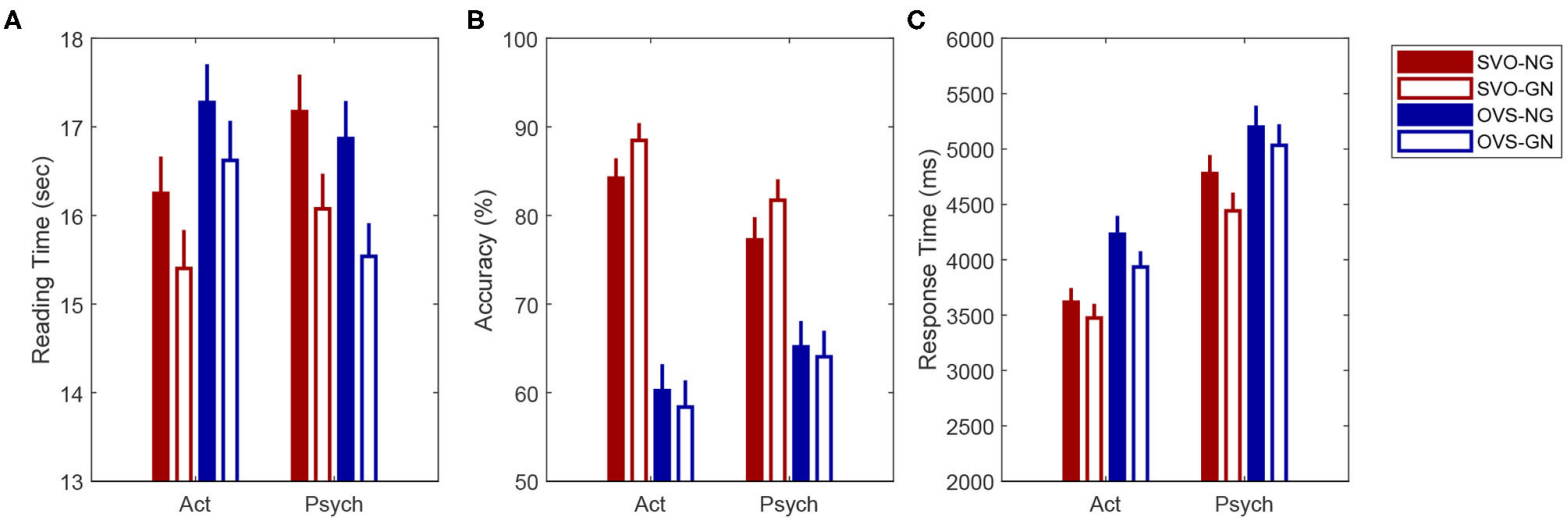
Mean total reading time for the critical texts **(A)**, percentage of accurate answers **(B)**, mean response times for the comprehension question **(C)**, in the current eye-tracking study according to verb type (ObjExp psych verb vs. Act), word order (SVO vs. OVS) and information structure (given-new vs. new-given). Error bars correspond to Standard Error of the Mean. ObjExp psych verb, Object Experiencer Psych Verb; Act, Activity Verb; SVO, Subject-Verb-Object; OVS, Object-Verb-Subject; GN, given-new; NG, new-given.

Figure 1A shows the average total reading time for the critical texts used in the current eye-tracking experiment. The statistical analysis revealed an interaction between verb type and word order; β = 0.022, *SE* = 0.007, *t* = 3.193, *p* < 0.01. Resolving this interaction showed that participants spent significantly longer time reading sentences with SVO word order when they included an ObjExp psych verb than when they included an activity verb; β = 0.071, *SE* = 0.020, *z* = 3.624, *p* < 0.01. Although this difference was not significant among OVS conditions, sentences with activity verbs were read slower when they followed the OVS word order than when they followed the SVO order; β = 0.077, *SE* = 0.020, *z* = 3.955, *p* < 0.001. Information structure also affected texts' reading times significantly. Participants took longer time to read texts in which the Information Structure of the critical sentence included a new referent in first position (*M* = 16,955 ms, *SE* = 210 ms) than when it included a given referent in first position (*M* = 16,015 ms, *SE* = 207 ms; β = 0.032, *SE* = 0.007, *z* = 4.552, *p* < 0.001).

#### 3.1.2. Question Accuracy

Mean accuracy for all comprehension questions was 86.27% (*SE* = 0.38%). This indicates that participants were paying attention to the content of the texts. Mean accuracy of critical questions was 77.45% (*SE* = 0.74%). Figure 1B shows mean accuracy according to condition. Differences in accuracy according to verb type and word order were analyzed with a generalized linear mixed-effects model. The analysis revealed a significant interaction between verb type and word order; β = −0.198, *SE* = 0.055, *z* = −3.614, *p* < 0.001. Resolving this interaction revealed that accuracy was significantly higher for questions about sentences with Activity verbs and SVO word order than for the other three conditions (ActSVO - ActOVS; β = 1.640, *SE* = 0.164, *z* = 9.981, *p* < 0.001; ActSVO - ObjExpSVO; β = 0.532, *SE* = 0.172, *z* = 3.089, *p* = 0.011; ActSVO - ObjExpOVS; β = 1.385, *SE* = 0.165, *z* = 8.397, *p* < 0.001). A significant effect of word order was also found. On average, participants responded more accurately after reading texts with sentences in SVO order (*M* = 84.51%, *SE* = 0.9) than texts with sentences in OVS order (*M* = 70.64%, *SE* = 1.13; β = −0.626, *SE* = 0.056, *z* = −11.27, *p* < 0.001).

#### 3.1.3. Response Time

Figure 1C shows mean response time (RT) according to condition. Analysis of differences in RT between verb type, word order and information structure revealed main effects of the three factors. On average, response time was significantly longer for questions about texts that included ObjExp psych verbs (*M* = 4,629 ms; *SE* = 70) than for questions about texts with activity verbs (*M* = 3,889 ms, *SE* = 58; β = −0.118, *SE* = 0.013, *t* = −9.066, *p* < 0.001). Participants also took longer time to respond to questions about texts that included sentences in OVS order (*M* = 4,396 ms; *SE* = 67) than when they included sentences in SVO order (*M* = 4,121 ms, *SE* = 61; β = 0.054, *SE* = 0.011, *t* = 5.031, *p* < 0.001). Finally, questions about texts that included critical sentences with non-canonical information structure were responded significantly slower (*M* = 4,383 ms; *SE* = 67) than questions about texts that included sentences with a canonical rhematic hierarchy (*M* = 4135 ms, *SE* = 62; β = 0.025, *SE* = 0.011, *t* = 2.270, *p* < 0.05). Interactions among the three factors were not significant.

### 3.2. Eyetracking Measures

**Figure 2:**
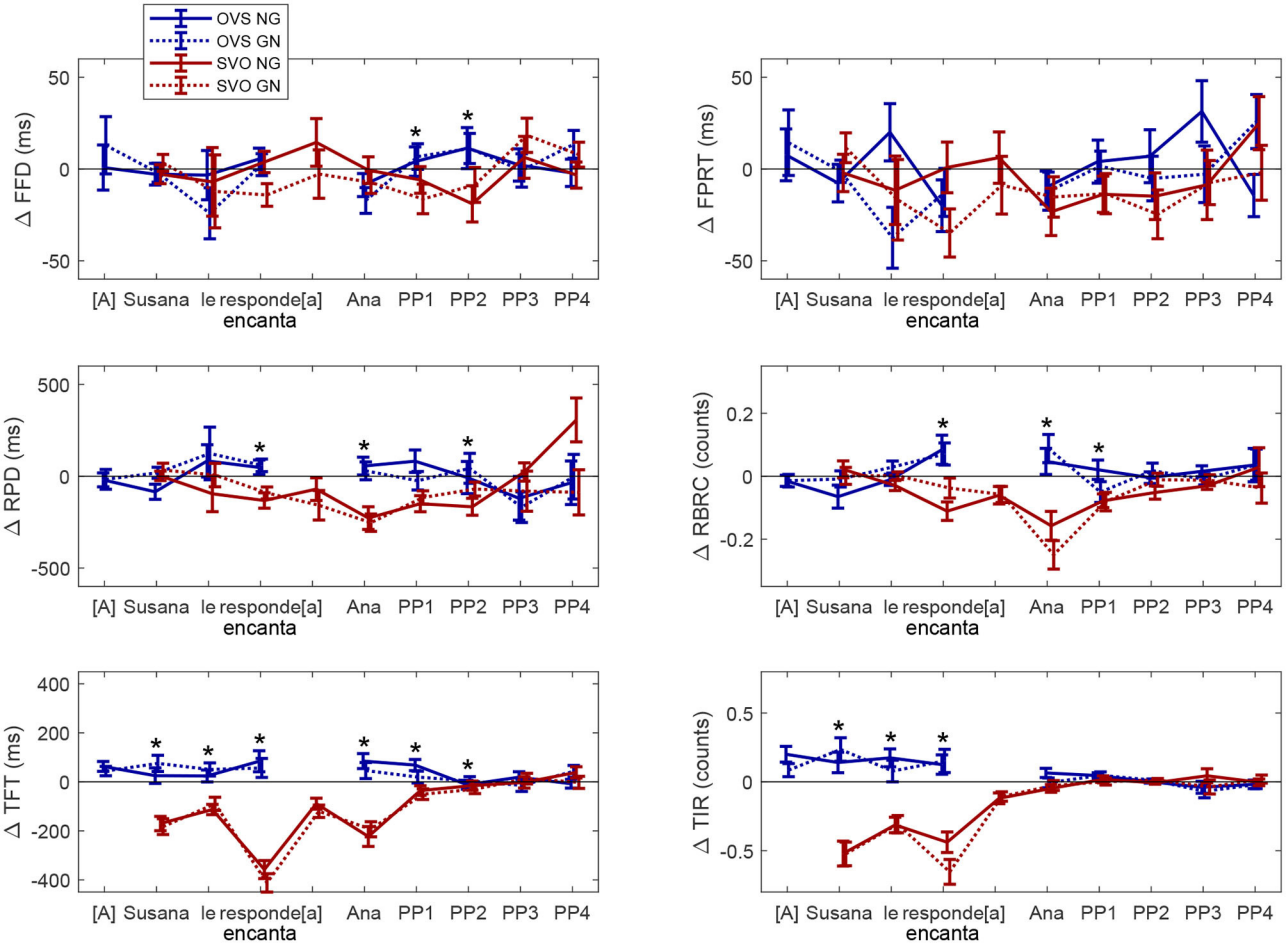
The Figure shows the difference (Δ) in mean fixation times (ms) and the amount of regressive saccades (counts) between conditions with Activity Verbs and conditions with Psych verbs according to the sentence word order (SVO vs. OVS) and information structure (Given-New vs. New-Given) Error bars correspond to Standard Error of the Mean. Eye-tracking measures: FFD, First Fixation Duration; FPRT, First Pass Reading Time; TFT, Total Fixation Time; RPD, Regression Path Duration; RBRC, Right-Bounded Regression Count; TIR, Total Incoming Regressions. Word Order: SVO, Subject-Verb-Object; OVS, Object-Verb-Subject. The asterisk shows that the interaction between Word Order and Verb Type was significant.

Figure 2 summarizes the contrast between sentences with activity verbs and sentences with ObjExp psych verbs according to both word orders (SVO in red and OVS in blue) and information structure (GN in dashed lines; NG in solid lines). Positive values mean that reading time is longer and regression counts are higher for sentences with activity verbs than for sentences with ObjExp psych verbs. A positive blue line and a negative red line correspond to an interaction between Verb Type and Word Order. Absolute values higher for solid lines than for dashed lines show an effect of Information Structure as expected, with non-canonical information structure showing higher cognitive demand than canonical information structure. This representation makes the interaction and Information Structure effect visually clear. The asterisks show the regions where the interaction was significant.

We now provide the analysis of regions of interest for both the early and late measures mentioned in the section 2.5. For each region, we first present the analysis of the interactions among factors since they comprise the contrasts of interest of the current study. We then provide the relevant results of the multiple comparisons test whenever was needed. Finally, we report main effects of Verb Type, Word Order or Information Structure. Appendix B shows the final converging models for each measure at each region. A table with all statistical coefficients, standard errors and *t* values may be found at Appendix C.

#### Region 1 (Case marking preposition “a”)

Analysis of late eye-movement measures revealed no interactions among factors. A main effect of Verb Type was found for Total Fixation Time (TFT) showing longer fixation time for sentences with activity verbs than for sentences with ObjExp psych verbs; β = 0.636, *SE* = 0.019, *t* = 3.311, *p* < 0.001.

#### Region 2 (First proper name)

Late eye-movement measures showed a significant interaction between Verb Type and Word Order for Total Fixation Time (TFT) and Total Incoming Regressions (TIR); TFT: β = 0.086, *SE* = 0.011, *t* = 7.923, *p* < 0.001; TIR: β = 0.192, *SE* = 0.018, *z* = 10.619, *p* < 0.001). Resolving these interactions revealed that for subject-initial sentences, the probability of regressing to this region and the total fixation time were significantly longer when the sentence included an ObjExp psych verb than when it included an activity verb (TFT: β = 0.259, *SE* = 0.031, *z* = 8.384, *p* < 0.001; TIR: β = 0.580, *SE* = 0.060, *z* = 9.590, *p* < 0.001). Conversely, for object-initial sentences, the probability of regressing into this region and the total fixation time were significantly longer when the sentence included an activity verb than when it included an ObjExp psych verb (TFT: β = 0.087, *SE* = 0.031, *z* = 2.811, *p* = 0.025; TIR: β = 0.181, *SE* = 0.056, *z* = 3.258, *p* = 0.005). Analysis of Regression Path Duration (RPD) also showed a main interaction between word order and information structure β =−0.027, *SE* = 0.0127, *t* = −2.158, *p* < 0.05. Tukey *post-hoc* test revealed that non-canonical word order (OVS) led to significantly longer regression path duration before continuing reading when the sentence presented a non-canonical information structure, β =−0.107, *SE* = 0.035, *z* = −3.022, *p* < 0.05. This difference was not significant between sentences with canonical information structure.

Analysis of early eye-movement measures revealed a main effect of Information Structure for First Fixation Duration (FFD) and First Pass Reading Time (FPRT), showing longer reading time for sentences with non-canonical Information Structure than for sentences with canonical Information Structure (FFD: β = −0.032, *SE* = 0.006, *t* = −5.018 , *p* < 0.001; FPRT: β = −0.052, *SE* = 0.008, *t* = −6.714,, *p* < 0.001.) A similar effect was found for Right-Bounded Regression Count (RBRC), showing higher amount of regressions from this region for sentences with non-canonical Information Structure: β = −0.016, *SE* = 0.039, *t* = −4.161, *p* < 0.01.

Analysis of the probability of regressions into this region (TIR), RBRC , RPD, and Total Fixation Time also revealed a main effect of Word Order. On average, participants regressed to this word significantly more, fixated on this word for longer time and regressed for significantly longer time and higher amount of times from this region in object-initial sentences than in subject-initial sentences; (TIR: β = 0.155, *SE* = 0.033, *z* = 4.710, *p* < 0.001; TFT: β = 0.063, *SE* = 0.011, *t* = 5.744, *p* < 0.001; RBRC: β = 0.010, *SE* = 0.040, *t* = 2.502, *p* < 0.05; RPD: β = 0.026, *SE* = 0.013, *t* = 2.065, *p* < 0.05 . Finally, effects of Verb Type and Information Structure were present for TFT and effects of Information Structure were found at RPD(TFT: Verb Type: β = −0.043, *SE* = 0.011, *t* = −3.963, *p* < 0.001; Information Structure:β = −0.110, *SE* = 0.013, *z* = −8.203, *p* < 0.001, RPD - Information Structure: *beta* = −0.083, *SE* = 0.013, *z* = −6.543, *p* < 0.001. The sign of these effects reveal that participants fixated for longer time when the sentence included an ObjExp Psych verb and fixated and regressed to previous regions for longer time when the noun corresponded to a new referent.

#### Region 3 (Clitic)

Analysis of early eye-movement measures revealed no interactions among fixed factors nor main effects. Analysis of late eye-movement measures showed a significant interaction between Verb Type and Word Order for total fixation time, and for the probability of regressions into this region (TFT: β = 0.074, *SE* = 0.014; *t* = 5.331; TIR: β = −0.218, *SE* = 0.024 *z* = 8.977, *p* < 0.001). This interaction follows the same direction as the interaction found on Region 2. For subject-initial sentences, participants regressed and fixated on this region significantly more when the sentence contained an ObjExp psych verb than when it included an activity verb (TFT: β = 0.226, *SE* = 0.040, *z* = 5.685, *p* < 0.001; TIR: β = 0.624, *SE* = 0.072, *z* = 8.630, *p* < 0.001). In object-initial sentences; participants regressed to this region significantly more when the sentence contained an activity verb; TIR: β = 0.246, *SE* = 0.067, *z* = 3.748, *p* < 0.001. This difference was not significant for TFT; β = 0.071, *SE* = 0.039, *z* = 1.817, *p* < 0.265. The analysis of these measures also revealed main effects of Verb Type (TFT: β = −0.039, *SE* = 0.014; *t* = −2.791; TIR: β = −0.094, *SE* = 0.024 *z* = −3.833, *p* < 0.01), Word Order (TIR: β = −0.075, *SE* = 0.024 *z* = 3.095, *p* < 0.01; RPD: β = 0.076 *SE* = 0.029, *t* = 2.639, *p* < 0.05), and Information Structure (TFT: β = −0.042, *SE* = 0.016; *t* = −2.597, *p* < 0.01). Participants showed higher processing load whenever the sentences included an ObjExp psych verb than when the included an activity verb. They also regressed to this region significantly more when the sentence followed the OVS order than when it followed the SVO word order, and fixated for longer time on this region when the first NP belonged to a new referent than when it belonged to an already given one.

#### Region 4 (Disambiguating verb)

Analysis of early eye-movement measures revealed no interactions among factors. However, a main effect of Information Structure was found for FPRT, showing significantly longer reading time for this region whenever the sentence presented a new referent in first position; β = −0.042, *SE* = 0.009, *t* = −4.706, *p* < 0.001. A significant interaction between Verb type and Word Order was found for all late eye-movement measures (RPD: β = 0.053, *SE* = 0.012, *t* = 4.449, *p* < 0.001; RBRC: β = −0.135, *SE* = 0.033, *z* = 4.081, *p* < 0.001; TFT: β = 0.119, *SE* = 0.010, *t* = 11.852tcr, *p* < 0.001; TIR: β = 0.202, *SE* = 0.019, *z* = 10.531, *p* < 0.001) Tukey *post-hoc* test showed that this interaction follows the same direction as in the previous region (SVO: RBRC: β = 0.276, *SE* = 0.102, *z* = 2.711, *p* < 0.05; TFT: β = 0.358, *SE* = 0.047, *z* = 7.701, *p* < 0.001; TIR: β = 0.631, *SE* = 0.081, *z* = 7.751, *p* < 0.001; OVS: RPD: β = 0.122, *SE* = 0.038, *z* = 3.222, *p* = 0.007; TFT: β = 0.118, *SE* = 0.046, *z* = 2.544, *p* < 0.05; RBRC: β = 0.266, *SE* = 0.091, *z* = 2.930, *p* < 0.05). TIR also showed a significant interaction among the three main factors, β = 0.041, *SE* = 0.019, *z* = 2.112, *p* < 0.05. Tukey HSD multiple comparisons showed that this triple interaction depended on the interaction between Verb and Word Order: when new information was in both first and second position, and sentences included an ObjExp psych verb, participants regressed significantly more to this region in SVO conditions than in OVS sentences (New-Given:β = 0.033, *SE* = 0.073, *z* = 4.546, *p* < 0.001; Given-New: β = 0.040, *SE* = 0.071, *z* = 5.641, *p* < 0.001). The opposite pattern took place for sentences with activity verbs: participants regressed significantly more to this region when the sentence followed the OVS word order than when it followed the SVO order (New-Given: β = 0.033, *SE* = 0.079, *z* = 4.160, *p* < 0.001; Given-New: β = 0.057, *SE* = 0.084, *z* = 6.762, *p* < 0.001). A significant effect of Word Order was found for RPD, RBRC and TFT in the same direction as in the previous region: participants found higher processing cost at this region for OVS sentences than for SVO sentences (RPD: β = 0.044, *SE* = 0.012, *t* = 3.739, *p* < 0.001; RBRC: β = 0.110, *SE* = 0.033, *z* = 3.280; TFT: β = 0.419, *SE* = 0.010, *t* = 4.173, *p* < 0.001) Furthermore, participants fixated for significantly longer time on this region and regressed significantly more to it whenever it included an ObjExp psych verb than when it included an activity verb (TFT: β = −0.060, *SE* = 0.021, *t* = −2.865, *p* < 0.01; TIR: β = −0.115, *SE* = 0.035, *z* = −3.251, *p* < 0.01). Finally, Information Structure modulated both RPD and TFT. Participants fixated for longer time on that region and previous regions before continuing reading and fixated for longer time on that word whenever the sentence included a new referent in first position (RPD: β = −0.062, *SE* = 0.012, *t* = −5.175, *p* < 0.001; TFT: β = −0.073, *SE* = 0.011, *t* = −6.827, *p* < 0.001).

#### Region 5 (Case marking preposition “a”)

Analysis of this region showed a significant main effect of Verb Type for most late eye-movement measures, with participants experiencing greater cognitive load and regressing significantly more to this region whenever the sentence included an ObjExp psych verb (RPD: β = −0.126, *SE* = 0.041; *t* = −3.074, *p* < 0.01; RBRC: β = −0.28617, *SE* = 0.089; *z* = −3.225, *p* < .001;TFT: β = −0.132, *SE* = 0.020; *t* = −6.544, *p* < 0.001; TIR: β = −0.348, *SE* = 0.059; *z* = −5.921, *p* < 0.010).

#### Region 6 (Second proper name)

Analysis of this region showed an interaction between Verb Type and Word Order for all late eye-movement measures (RPD: β = 0.116, *SE* = 0.015, *t* = 7.598, *p* < 0.001; RBRC: β = 0.177, *SE* = 0.028, *z* = 6.253, *p* < 0.01; TFT: β = 0.010; *SE* = 0.011; *t* = 9.150, *p* < 0.001; TIR: β = 0.084; *SE* = 0.040; *t* = 2.125, *p* < 0.05). Resolving these interactions revealed that in SVO sentences, participants fixated for longer time at this and previous regions and regressed significantly more times from and to this region when the sentence contained an ObjExp psych verb than when it included an activity verb (RPD: β = 0.398, *SE* = 0.043, *z* = 9.235, *p* < 0.001; RBRC: β = 0.534, *SE* = 0.081, *z* = 6.573, *p* < 0.001; TFT: β = 0.332, *SE* = 0.033, *z* = 9.962, *p* < 0.001; TIR: β = 0.624, *SE* = 0.072, *z* = 8.693, *p* < 0.001).

Difference among OVS conditions was only significant for Total Incoming Regressions, with conditions with activity verbs showing a higher amount of regressions to this region than conditions with ObjExp psych verbs (β = 0.246, *SE* = 0.065, *z* = 3.777, *p* < 0.001).

A main effect of Word Order was found for FFD and FPRT, with longer reading times for sentences with SVO word order than for sentences with OVS order (FFD: β = −0.016; *SE* = 0.007; *t* = −2.292, *p* = 0.002; FPRT: β = −0.034; *SE* = 0.008; *t* = −3.989, *p* < 0.01).

A significant effect of Verb Type was found for FPRT and all late eye-movement measures except for TIR. The sign of the effect shows longer reading time, regression duration and amount of regressions from this region when the sentences included an ObjExp psych verb than when they included an activity verb (FPRT: β = −0.023; *SE* = 0.008; *t* = −2.724, *p* < 0.01; RPD: β = −0.083; *SE* = 0.015; *t* = −5.438, *p* < 0.001; RBRC: β = −0.090; *SE* = 0.028; *z* = −3.180, *p* < 0.01; TFT: β = −0.064; *SE* = 0.012; *t* = −5.172). Finally, a significant effect of Information Structure was also for FPRT. Contrary to the effect of Information Structure found in previous regions, this region shows longer reading time for conditions with new information in second position than for conditions with new information in first position; β = −0.026; *SE* = 0.008; *t* = −3.101, *p* < 0.01.

#### Region 7 (First word of the Spill-over region)

Analysis of this region showed that the interaction between Verb Type and Word Order was significant for FFD and for three out of five late eye-movement measures (FFD: β = 0.020, *SE* = 0.008, *t* = 2.649, *p* = 0.008; RPD: β = 0.066, *SE*= 0.016, *t* = 4.206, *p* < 0.001; RBRC: β = 0.151; *SE* = 0.051, *z*=2.953, *p* < 0.05; TFT: β = 0.052; *SE* = 0.011, *t* = 4.579, *p* < 0.001). The multiple comparisons test showed that in subject-initial sentences, participants fixated for longer time at this region when the sentence contained an ObjExp psych verb than when it included an activity verb (TFT: β = 0.0332, *SE* = 0.032, *t* = 10.477, *p* < 0.001). Participants also spent longer time reading and regressing to previous regions and regressed significantly more times from this region for sentences with ObjExp psych verbs than for sentences with activity verbs (RPD: β = 0.021, *SE* = 0.045, *z* = 4.724, *p* < 0.001; RBRC: β = 0.665; *SE* = 0.146, *t* = 4.551, *p* < 0.001).

Differences among object-initial sentences were marginally significant for Total Fixation Time, with participant fixating for longer time on this region when the sentence included an activity verb than when it included an ObjExp psych verb; TFT: β = 0.077, *SE* = 0.032, *z* = 2.436, *p* = 0.070. For the other four measures this difference was not significant.

Analysis of this region also showed main effects of word order and verb type for late measures RPD and RBRC. Participants regressed significantly more from this region and spent significantly longer time on previous regions before continuing reading when the sentences included ObjExp psych verbs than when they included activity verbs and when they followed the OVS word order than when they were subject-initial sentences (Word Order: RPD: β = 0.049, *SE* = 0.016, *t* = 3.127, *p* < 0.01; RBRC: β = 0.021, *SE* = 0.048, *z* = 4.454, *p* < 0.001; Verb: RPD: β = −0.039, *SE* = 0.016, *t* = −2.517, *p* < 0.05; RBRC: β = −0.184, *SE* = 0.046, *z* = −3.983, *p* < 0.01).

#### Region 8 (Second word of the Spill-over Region)

A significant interaction between Verb Type and Word Order was found for FFD and RPD (FFD: β = 0.023, *SE* = 0.010, *t* = 2.555, *p* < 0.05; RPD: β = 0.042, *SE* = 0.019, *t* = 2.224, *p* < 0.05). The multiple comparisons Tukey HSD test revealed significant differences among SVO conditions for RPD only, with conditions with ObjExp psych verbs engendering longer regression path duration than sentences with activity verbs. Differences among OVS conditions were not significant for any of the above-mentioned measures. Analysis of FFD and TFT also showed a significant interaction between Word Order and Information Structure (FFD: β = 0.021, *SE* = 0.010, *t* = 2.087, *p* < 0.05; TFT: β = 0.028, *SE* = 0.013, *t* = 2.138). However, the interaction was not confirmed by the multiple comparisons tests from both measures. Finally, a main effect of Verb Type was found for RPD, with sentences with ObjExp psych verbs engendering longer regression path duration than sentences with activity verbs; β = −0.046, *SE* = 0.019, *t* = −2.413, *p* < 0.05.

#### Region 9 (Third word of the Spill-over Region)

Analysis of early eye-movement measures revealed a significant interaction between Word Order and Information Structure for FPRT; β = 0.021, *SE* = 0.010, *t* = 2.037, *p* < 0.05. However, the multiple comparisons test showed no significant differences among conditions.

Analysis of late eye-movement measures showed a main effect of Verb Type for RPD and a main effect of Word Order for TIR (Verb: β = −0.055, *SE* = 0.026, *t* = −2.113, *p* < 0.05; Word Order: β = 0.056, *SE* = 0.026, *z* = 2.198, *p* < 0.05). The sign of the effects show that participants regressed significantly longer time to previous regions when the sentence included an ObjExp psych verb and that they regressed more times to this region when the sentence followed the OVS word order.

#### Region 10 (Fourth word of the Spill-over Region)

Analysis of this region showed a significant interaction among Word Order, Verb Type and Information Structure for FPRT and RPD (FPRT: β = 0.024, *SE* = 0.010, *t* = 2.484, *p* < 0.05; RPD: β = 0.056, *SE* = 0.021, *t* = 2.680, *p* < 0.01). Resolving these interactions revealed that for First Pass Reading Time, participants spent longer time reading this region in sentences with activity verbs and a new referent in first position when the sentence followed the SVO order than when it followed the OVS one; β = 0.122; *SE* = 0.039, *t* = 3.164, *p* < 0.05. Differences among the other conditions did not reach significance for this measure nor for RPD. A main effect of Word Order was also sound for FPRT. Participants spent longer time on this region when word order was SVO than when it was OVS, β =−0.0239, *SE* = 0.010, *t* = −2.486 , *p* < 0.05 Finally, a main effect of Information Structure was found for RPD, β =−0.064, *SE* = 0.021, *t* = −3.078, *p* < 0.01. The sign of the effect shows longer reading time for conditions with new information in second position than for conditions with new information in first position.

## 4. Discussion

Evidence about the comprehension of isolated Spanish sentences with alternative word orders has shown that readers manifest increasing difficulty to understand sentences with a word order that does not respect the order of arguments at the lexico-semantic structure of the verb, independently of whether the sentence follows the canonical word order of the language (SVO) or not (Gattei et al., 2015a,b, 2017). In these studies, the authors used sentences with activity verbs and object experiencer verbs in order to compare events that required alternative linking between syntax and semantics. When reading SVO sentences, participants required significantly more time to read and figure out “who did / felt what for whom” when the sentence included an ObjExp psych verb. Conversely, when reading OVS sentences, participants required more time to read when the sentence included an activity verb. This interaction was present when using both self-paced reading and eye-tracking techniques.

These studies support the hypothesis that during incremental parsing, readers use the morphosyntactic and semantic information provided by the first sentential argument to generate predictions about the verb type that will take place in the sentence according to the prominence status of that argument. This proposal assumes that the language processing system gives rise to predictions about arguments order by following a prominence hierarchy that canonically stipulates that more prominent arguments precede less prominent ones (Bornkessel et al., 2005; Wolff et al., 2007; Haupt et al., 2008). Nonetheless, it is relevant to ask whether the pragmatic use of constituents order has any influence on the mentioned results. In the current study, we focused on the distinction between “given” and “new” referents in a sentence in relation with a previous context. According to Givón (1984), the use of a non-canonical word order is expected when mentioning a referent that has already been introduced by the previous context so that the rhematic hierarchy (i.e., given-new) is respected. Experimental evidence about the role of rhematic hierarchy during incremental reading has shown that effects of word order non-canonicity are alleviated when an adequate context precedes the sentence (Kaiser and Trueswell, 2004; Burmester et al., 2014), suggesting that increasing reading times in isolated sentences with non-canonical word order could partly be due to higher cognitive demands related to making assumptions about possible contexts in which a non-canonical word order could take place. However, the role of information structure in relation to alternative word orders stipulated by different lexico-semantic configurations had not been explored yet. Thus, the question that motivated the present study was whether the prominence effects found for Spanish sentence comprehension were caused by the lack of a context that could motivate the election of a specific word order. We thus framed sentences used in Gattei et al. (2017) in texts that would favor the appearance of a specific referent in first position of the sentence and compared them with sentences in which the first argument comprised a new referent. By means of a comprehension offline task, we also evaluated the cognitive cost of understanding “who did/felt what for whom” correctly.

Regarding the hypotheses and predictions outlined at the section 1, the current work shows that context adequacy plays a role for processing of sentences with non-canonical word order but does not make effects of prominence miscomputation fade away.

On the one hand, results of the current study revealed that the use of an adequate context facilitated the comprehension of the target sentences. Participants took significantly less time to read the texts when, in first position, the final sentence introduced a referent that had explicitly been presented before. They also took less time to respond the comprehension questions when the target sentence included a canonical information structure. In other words, these results replicate the findings that Kaiser and Trueswell (2004) showed for Finnish sentences with activity verbs. Additionally, the current study revealed that a non-canonical information structure is detrimental to comprehension even in SVO sentences, as it is evidenced by sentences' response time of questions about sentences with ObjExp psych verbs.

On the other hand, effects related to incorrect syntax-to-semantic linking were present during reading for late eye-movement measures as predicted, showing a disruption of processes of higher-level text integration (Clifton et al., 2007). When encountering a verb that did not match the predictions stipulated by the computation of prominence status of the first argument, participants took longer time to read the word and following content words, and regressed more times and for longer time to previous regions. These results replicate the findings by Gattei et al. (2017) for isolated sentences, and yield further evidence in favor of the hypothesis that one of the central mechanisms for argument interpretation is prominence computation (Bornkessel and Schlesewsky, 2006), and that prominence computation follow a principle that assumes that the first argument will be the most “Actor-like” possible.

The current study also provides interesting insight about the cognitive cost and strategies used by readers to process new information. Several proposals have tried to explain the cognitive effects derived from the use of a non-canonical rhematic hierarchy. Although there is an agreement regarding the type of effects caused by the unpredictable appearance of a new referent, there is not a unique view with respect to which mechanisms are involved in information structure processing. For instance, it has been proposed that speakers tend to choose the syntactic constructions that allow them to place the most “accessible” (already mentioned) information earlier in the utterance (Ferreira, 2003), possibly because this allows them to postpone the difficult part of the utterance, which requires more resources to plan. The assumption behind this hypothesis is that when information has a strong representation in memory it is easier to retrieve and to process. Evidence in favor of this view shows that speakers choose word order according to visual attention (Gleitman et al., 2007).

Kaiser (2012) argues that the pragmatic status of referent emerges naturally from memory and attention. Theories about memory distinguish between working memory, which stores information currently being used, and long-term memory, which stores the conceptual and procedural knowledge for posterior use. From this point of view, given referents can be defined as those accessed through working memory (and thus easier to retrieve) and new referents as those which have not been retrieved by long-term memory yet (Arnold et al., 2013).

From a neurobiological perspective of language and its processing, Bornkessel-Schlesewsky and Schumacher (2016) propose that, instead of postulating specific neural correlates for information structure, a more promising approach is to consider that information structure affects domain-general mechanisms when hierarchically guiding predictive processing or when providing cues for attentional shift. The authors claim that the status of discourse referents feeds the predictive processes during discourse, as it is shown by how the preference for a continuity of the same referent or for certain types of linearization (i.e., given referents precede new ones) facilitate language processing. Errors in the predictions at this level elicit negative potentials (for instance N400 for unpredictable information structure properties) and result in attentional reorienting and mental model updating required in topic shift scenarios.

A general idea that stems from these approaches is that the violation of rhematic structure involves the modification of a more flexible system, designed to adapt linguistic form to the dynamics of context -possibly through working memory capacity-, and prepared to deal with domain-general mechanisms like attentional shift and reorienting. Although the current work was not aimed at disentangle whether the effects produced by the use of a non-canonical rhematic hierarchy were related to factors associated to referents' accessibility, readers' working memory capacity or a failure of an expected structure and the subsequent need for attentional reorientation, the current findings are informative with regards to the mechanisms underlying incremental processing of new information.

In the current study, eye-movement measures showed information structure effects, evidenced by increasing reading times for the first and the second NP whenever readers found a new referent. However, it is generally argued that reading words that are repeated throughout a text entails a decrease of reading time (Rayner et al., 1995; O'Brien et al., 1997; Kamienkowski et al., 2018). Hence, it is fair to ask whether the effects found at new referents respond to the manipulation of rhematic hierarchy or if they should be interpreted as lexical repetition effects related to word recognition processes. For instance, Lowder et al. (2013) ran en eye-tracking study in which participants had to read sentences with two NPs composed by two and one proper names, respectively. The authors manipulated proper names' frequency and repetition (the second NP could mention one of the proper names from the first NP or not) and showed that when reading the second NP, repeated names were processed more quickly than new names in both early and late eye-movement measures. Following Gordon and Hendrick (1998), the authors argue that “while basic word recognition goes on, the effort to understand the meaning of a sentence or short discourse leads to the construction of a discourse model that represents patterns of reference and co-reference and which captures the predicate-argument relationships described in the text” (Lowder et al., 2013). The results of the current study showed a similar pattern of results, with a modulation of both early and late eye-movement measures at the first NP and following two regions, when the proper name comprised a new referent. Interestingly, when the sentence followed a canonical information structure, effects of a new referent were only present for First Pass Reading Time at the proper name region. We interpret this pattern of results as a difference in the control and time course of oculomotor processes for word recognition, with short-lived, early effects, and for information structure manipulation, which affected late eye-movements and caused a longer comprehension disruption.

As for offline measures collected in the current study, total reading time is informative of the time required by readers to guarantee that they have understood the text. Although these were the instructions provided, this measure did not reflect comprehension success, as shown by accuracy rates. In particular, participants responded questions significantly better when the final sentence followed Spanish canonical word order (SVO), independently of whether the initial constituent consisted of a new referent or not. Although this was expected for sentences with activity verbs, a preference for SVO word order was not expected for accuracy rates of sentences with ObjExp psych verbs, diverging from the results found in previous studies about this issue with isolated sentences. In Gattei et al. (2017) participants showed overall higher accuracy rates (around 90% accuracy for critical questions), and higher accuracy rates for questions about sentences with activity verbs than for those about ObjExp psych verbs. While further investigation of this difference between experiments is needed, it is possible that the use of additional context and the requirement of keeping referents in working memory in order to reply the comprehension questions, had a negative effect on the comprehension of Spanish overall less frequent word order.

With regards to the interaction between verb type and word order found for accuracy rates, results replicate the findings of Gattei et al. (2017), with higher accuracy for questions about SVO sentences with activity verbs than for the other conditions. We argue that this pattern is expected as in this type of sentences both semantic and syntactic canonical orders coincide, while the other conditions present an alteration of either semantic order (as in SVO sentences with ObjExp psych verbs), constituents order (as in OVS sentences with ObjExp psych verbs) or both (as in OVS sentences with Activity verbs). In other words, results show that the alignment of both canonical linking and canonical word constituents order facilitates comprehension, whereas non canonical arrangement of either type of information makes it more difficult.

A final aspect that needs to be taken into account is the response time for comprehension questions, which show that readers needed extra time to respond questions about sentences with either OVS word order, non-canonical information structure or ObjExp psych verbs. We believe that the lack of information structure effects for overall accuracy shows that while the use of a non-canonical rhematic hierarchy require longer reading time and response time for comprehension questions, the consequences of not following a canonical order for information structure are not as strong as to show a modulation of comprehension success, as it occurs with OVS sentences. However, it is matter of future research to evaluate whether differences in the trade-off between response time and accuracy for word order and information structure non-canonicity respond to task-related factors (as structural complexity or types of questions used) or individual differences (like working memory or attentional capacities).

### 4.1. Possible Methodological Caveats and Future Directions

Although results of the current study support previous results on this issue, possible methodological caveats should be taken into account for future research and replication in other languages. Most importantly, while the study asks about the role of prominence computation in sentences embedded in context, the materials were designed in such way that they do not directly compare comprehension of isolated sentences with comprehension of sentences embedded in texts within the same group of subjects. The rationale of not doing so was that adding no-context trials would have implied to double the amount of conditions to sixteen conditions. Considering the short amount of ObjExp psych verbs available in Spanish, this would have implied that participants either read each verb four times (as opposed to two as it occurs in the current version of the experiment), enabling the possibility of introducing the confound of structure repetition effect and other possible confounds due to participants tiredness or boredom, or that the amount of subjects tested was doubled to approximately 150 to yield results comparable to the current ones. Considering that the sentences without context have been repeatedly tested (Gattei et al., 2015a,b, 2017) we considered that the design of the current study was a fair trade-off between running the ideal experiment and getting reliable results. Still, adding further isolated conditions in languages that have not been previously tested would be crucial for results' replication.

A second aspect that needs further investigation is how participants deal with referent's activation when encountering sentences with non-canonical information structure. In other words, can regressive saccades from regions comprising new referents to previous sentences be informative of participants' reading strategies related to referent updating? (Chafe, 1976, 1994). While, this question was out of the scope of our work, we are currently addressing this issue with the data currently collected.

## 5. Conclusions

We presented an exploratory study that evaluated the interaction between word order, lexico-semantic structure of the verb and information structure in the comprehension of Spanish texts. Previous studies about this language have only evaluated the role of the first two factors, leaving aside the pragmatic aspect involved in the election of constituents' word order. Understanding the role of information structure is crucial to explain sentence processing in this language, since previous evidence has shown that when sentences are presented in isolation, constituents order is a relevant cue for incremental argument interpretation. It was pertinent to ask whether word order is still a relevant cue for argument interpretation when the previous context justifies (or not) the appearance of a specific word order. By evaluating reading of texts that manipulated the relation between “given” and “new” referents we showed that while information structure canonicity enhances comprehension, the use of an adequate context for a specific word order does not alleviate comprehension effects caused by argument misinterpretation. This type of evidence is crucial for any model of language comprehension that attempts to explain sentence processing in languages that allow alternative word orders.

By conducting an eye-tracking experiment, we could also provide further information about the time course of on-going processing of new referents, which show a different gaze signature to lexically-driven word retrieval.

## Data Availability Statement

The raw data supporting the conclusions of this article are available at https://osf.io/kp4dn/.

## Ethics Statement

The studies involving human participants were reviewed and approved by Facultad de Filosofía y Letras - Universidad de Buenos Aires. The participants provided their written informed consent to participate in this study.

## Author Contributions

CG designed the materials of the study, collected the data, ran the statistical analysis, and sketched the first draft of this manuscript. DS programmed the Matlab script for data collection, data preanalysis, and figures' design. LP supervised the adequacy of hypotheses and predictions to formal approaches of information structure canonicity. All authors equally contributed with the initial discussion of hypotheses, predictions and results of the current study, and writing the final version of this manuscript.

## Conflict of Interest

The authors declare that the research was conducted in the absence of any commercial or financial relationships that could be construed as a potential conflict of interest.

## References

[B1] Arnold J. E. Kaiser E. Kahn J. M. Kim L. K. (2013). Information structure: linguistic, cognitive, and processing approaches. Wiley Interdiscipl. Rev. Cogn. Sci. 4, 403–413. 10.1002/wcs.1234 PMC4491328 26150905

[B2] Baayen R. H. (2008). Analyzing Linguistic Data: A Practical Introduction to Statistics Using R. Cambridge, MA: Cambridge University Press. 10.1017/CBO9780511801686

[B3] Bader M. Bayer J. (2006). Case and Linking in Language Comprehension: Evidence from German, Volume 34 of Studies in Theoretical Psycholinguistics. Dordrecht: Springer Science & Business Media.

[B4] Bader M. Meng M. (1999). Subject-object ambiguities in German embedded clauses: an across-the-board comparison. J. Psycholinguist. Res. 28, 121–143. 10.1023/A:1023206208142

[B5] Barr D. J. Levy R. Scheepers C. Tily H. J. (2013). Random effects structure for confirmatory hypothesis testing: keep it maximal. J. Mem. Lang. 68, 255–278. 10.1016/j.jml.2012.11.001 24403724 PMC3881361

[B6] Bates D. Machler M. Bolker B. Walker S. (2014). Fitting linear mixed-effects models using lme4. arXiv preprint arXiv:1406.5823. 10.18637/jss.v067.i01

[B7] Belloro V. (2012). “La estructura informativa,” in El funcionalismo en la teoría lingüística: la Gramática del Papel y la Referencia; Introducción, avances y aplicaciones, eds R. Mairal, L. Guerrero, and C. González Vergara (Madrid: Akal), 225–244.

[B8] Birner B. J. Ward G. L. (1998). Information Status and Noncanonical Word Order in English, Vol. 40. Amsterdam: John Benjamins Publishing. 10.1075/slcs.40

[B9] Bornkessel I. Schlesewsky M. (2006). The extended argument dependency model: a neurocognitive approach to sentence comprehension across languages. Psychol. Rev. 113:787. 10.1037/0033-295X.113.4.787 17014303

[B10] Bornkessel I. Schlesewsky M. Friederici A. D. (2003). Eliciting thematic reanalysis effects: the role of syntax-independent information during parsing. Lang. Cogn. Process. 18, 269–298. 10.1080/01690960244000018

[B11] Bornkessel I. Zysset S. Friederici A. D. von Cramon D. Y. Schlesewsky M. (2005). Who did what to whom? The neural basis of argument hierarchies during language comprehension. Neuroimage 26, 221–233. 10.1016/j.neuroimage.2005.01.032 15862222

[B12] Bornkessel-Schlesewsky I. Schumacher P. B. (2016). “Towards a neurobiology of information structure,” in The Oxford Handbook of Information Structure, eds C. Fery and S. Ishihara (Oxford: Oxford University Press), 581–598. 10.1093/oxfordhb/9780199642670.013.22

[B13] Burmester J. Spalek K. Wartenburger I. (2014). Context updating during sentence comprehension: the effect of aboutness topic. Brain Lang. 137, 62–76. 10.1016/j.bandl.2014.08.001 25156161

[B14] Chafe W. (1976). “Givenness, contrastiveness, definiteness, subjects, topics, and point of view,” in Subject and Topic, ed C. Li (New York, NY: Academic Press).

[B15] Chafe W. (1994). Discourse, Consciousness, and Time: The Flow and Displacement of Conscious Experience in Speaking and Writing. University of Chicago Press.

[B16] Chow W.-Y. Phillips C. (2013). No semantic illusions in the 'semantic P600' phenomenon: ERP evidence from Mandarin Chinese. Brain Res. 1506, 76–93. 10.1016/j.brainres.2013.02.016 23422676

[B17] Clifton C. Staub A. Rayner K. (2007). “Eye movements in reading words and sentences,” in Eye Movements: A Window on Mind and Brain, ed R. van Gompel (Elsevier Science), 341–372. 10.1016/B978-008044980-7/50017-3

[B18] Comrie B. (1989). Language Universals and Linguistic Typology: Syntax and Morphology. University of Chicago Press.

[B19] Contreras H. (1976). A Theory of Word Order with Special Reference to Spanish. Amsterdam: North-Holland.

[B20] Cornelissen F. W. Peters E. M. Palmer J. (2002). The eyelink toolbox: eye tracking with MATLAB and the psychophysics toolbox. Behav. Res. Methods Instrum. Comput. 34, 613–617. 10.3758/BF03195489 12564564

[B21] Davis C. J. Perea M. (2005). Buscapalabras: a program for deriving orthographic and phonological neighborhood statistics and other psycholinguistic indices in Spanish. Behav. Res. Methods 37, 665–671. 10.3758/BF03192738 16629300

[B22] Dröge A. Maffongelli L. Bornkessel-Schlesewsky I. (2014). “Luigi piace a Laura?” in Structuring the Argument: Multidisciplinary Research on Verb Argument Structure, eds A. Bachrach, I. Roy, and L. Stockall (Amsterdam: John Benjamins Publishing Company), 83–118. 10.1075/lfab.10.05dro

[B23] Ferreira V. S. (2003). The persistence of optional complementizer production: why saying “that” is not saying “that” at all. J. Mem. Lang. 48, 379–398. 10.1016/S0749-596X(02)00523-5

[B24] Foley W. A. Van Valin R. D. Jr. (1984). Functional Syntax and Universal Grammar, Volume 38 of Cambridge Studies in Linguistics. London: Cambridge University Press.

[B25] Gattei C. A. Dickey M. W. Wainselboim A. J. París L. (2015a). The thematic hierarchy in sentence comprehension: a study on the interaction between verb class and word order in Spanish. Q. J. Exp. Psychol. 68, 1981–2007. 10.1080/17470218.2014.1000345 25529525

[B26] Gattei C. A. Sevilla Y. Tabullo A. Wainselboim A. París L. Shalom D. (2017). Prominence in spanish sentence comprehension: an eye-tracking study. Lang. Cogn. Neurosci. 33, 587–607. 10.1080/23273798.2017.1397278

[B27] Gattei C. A. Tabullo Á. París L. Wainselboim A. J. (2015b). The role of prominence in Spanish sentence comprehension: an ERP study. Brain Lang. 150, 22–35. 10.1016/j.bandl.2015.08.001 26291770

[B28] Givón T. (1984). Syntax: A Functional-Typological Approach. Amsterdam: J. Benjamins. 10.1075/z.17

[B29] Gleitman L. R. January D. Nappa R. Trueswell J. C. (2007). On the give and take between event apprehension and utterance formulation. J. Mem. Lang. 57, 544–569. 10.1016/j.jml.2007.01.007 18978929 PMC2151743

[B30] Gordon P. C. Hendrick R. (1998). The representation and processing of coreference in discourse. Cogn. Sci. 22, 389–424. 10.1207/s15516709cog2204_1

[B31] Haupt F. S. Schlesewsky M. Roehm D. Friederici A. D. Bornkessel-Schlesewsky I. (2008). The status of subject-object reanalyses in the language comprehension architecture. J. Mem. Lang. 59, 54–96. 10.1016/j.jml.2008.02.003

[B32] Hyönä H. Hujanen J. (1997). Effects of case marking and word order on sentence parsing in finnish: an eye fixation analysis. Q. J. Exp. Psychol. A 50, 841–858. 10.1080/027249897391919

[B33] Just M. A. Carpenter P. A. (1980). A theory of reading: from eye fixations to comprehension. Psychol. Rev. 87:329. 10.1037/0033-295X.87.4.329 7413885

[B34] Just M. A. Carpenter P. A. Woolley J. D. (1982). Paradigms and processes in reading comprehension. J. Exp. Psychol. Gen. 111:228. 10.1037/0096-3445.111.2.228 6213735

[B35] Kaiser E. (2012). Taking action: a cross-modal investigation of discourse-level representations. Front. Psychol. 3:156. 10.3389/fpsyg.2012.00156 22701440 PMC3372065

[B36] Kaiser E. Trueswell J. C. (2004). The role of discourse context in the processing of a flexible word-order language. Cognition 94, 113–147. 10.1016/j.cognition.2004.01.002 15582623

[B37] Kamide Y. Mitchell D. C. (1999). Incremental pre-head attachment in Japanese parsing. Lang. Cogn. Process. 14, 631–662. 10.1080/016909699386211

[B38] Kamienkowski J. E. Carbajal M. J. Bianchi B. Sigman M. Shalom D. E. (2018). Cumulative repetition effects across multiple readings of a word: evidence from eye movements. Discourse Process. 55, 256–271. 10.1080/0163853X.2016.1234872

[B39] Kliegl R. Grabner E. Rolfs M. Engbert R. (2004). Length, frequency, and predictability effects of words on eye movements in reading. Eur. J. Cogn. Psychol. 16, 262–284. 10.1080/09541440340000213

[B40] Lambrecht K. (1994). Information Structure and Sentence Form: Topic, Focus, and the Mental Representations of Discourse Referents, Vol. 71. Cambridge, MA: Cambridge University Press. 10.1017/CBO9780511620607

[B41] Lamers M. De Swart P. (2012). Case, Word order and Prominence: Interacting Cues in Language Production and Comprehension, Vol. 40. Dordrecht: Springer Science & Business Media. 10.1007/978-94-007-1463-2

[B42] Logacev P. Vasishth S. (2013). em2: A Package for Computing Reading Time Measures for Psycholinguistics. R package version *0.9*. Available online at: http://CRAN.R-project.org/package=em2

[B43] Lowder M. W. Choi W. Gordon P. C. (2013). Word recognition during reading: the interaction between lexical repetition and frequency. Mem. Cogn. 41, 738–751. 10.3758/s13421-012-0288-z 23283808 PMC3632652

[B44] O'Brien E. J. Raney G. E. Albrecht J. E. Rayner K. (1997). Processes involved in the resolution of explicit anaphors. Discourse Process. 23, 1–24. 10.1080/01638539709544979

[B45] Ocampo F. (1995). “The word order of two-constituent constructions in spoken Spanish,” in Word Order in Discourse, eds P. A. Downing and M. Noonan (Amsterdam: John Benjamins), 425–447. 10.1075/tsl.30.15oca

[B46] Pinheiro J. C. Bates D. M. (2000). “Linear mixed-effects models: basic concepts and examples,” in Mixed-Effects Models in S and S-Plus, eds J. C. Pinheiro and D. M. Bates (New York, NY: Springer), 3–56. 10.1007/978-1-4419-0318-1_1

[B47] R Core Team (2013). R: A Language and Environment for Statistical Computing. Vienna: R Foundation for Statistical Computing.

[B48] Rayner K. Raney G. E. Pollatsek A. (1995). “Eye movements and discourse processing,” in Sources of Coherence in Reading, eds R. F. Lorch Jr., and E. J. O'Brien (Hillsdale), 9–35.

[B49] Rayner K. Well A. D. (1996). Effects of contextual constraint on eye movements in reading: a further examination. Psychon. Bull. Rev. 3, 04–509. 10.3758/BF03214555 24213985

[B50] Schumacher P. B. Hung Y.-C. (2012). Positional influences on information packaging: insights from topological fields in German. J. Mem. Lang. 67, 295–310. 10.1016/j.jml.2012.05.006

[B51] Suñer M. (1982). Syntax and Semantics of Spanish Presentational Sentence-Types. Georgetown University School of Language.

[B52] Van Valin R. D. LaPolla R. J. (1997). Syntax: Structure, Meaning, and Function. Cambridge, MA: Cambridge University Press. 10.1017/CBO9781139166799

[B53] Van Valin R. D. Jr. (1999). “A typology of the interaction of focus structure and syntax,” in Typology and the Theory of Language: From Description to Explanation, eds E. Raxilina and J. Testelec (Moscow: Languages of Russian Culture), 511–524.

[B54] Van Valin R. D. Jr. (2005). Exploring the Syntax-Semantics Interface. Cambridge, MA: Cambridge University Press. 10.1017/CBO9780511610578.001

[B55] Vasishth S. von der Malsburg T. Engelmann F. (2013). What eye movements can tell us about sentence comprehension. Wiley Interdiscipl. Rev. Cogn. Sci. 4, 125–134. 10.1002/wcs.1209 26304190

[B56] Wang L. Schlesewsky M. Philipp M. Bornkessel-Schlesewsky I. (2012). “The role of animacy in online argument interpretation in Mandarin Chinese,” in Case, Word Order and Prominence, eds M. Lamers and P. de Swart (Dordrecht: Springer), 91–119. 10.1007/978-94-007-1463-2_5

[B57] Wolff S. Schlesewsky M. Bornkessel-Schlesewsky I. (2007). The interaction of universal and language-specific properties in the neurocognition of language comprehension: evidence from the processing of word order permutations in Japanese. J. Cogn. Neurosci 288.

[B58] Zubizarreta M. L. (1998). Prosody, Focus, and Word Order. Cambridge, MA: MIT Press.

